# 

**Published:** 1902-03-22

**Authors:** 


### IMPORTANT NOTICE.

**On account of the Easter Holidays, we beg to announce that we shall go to press with our issue of the 29th MARCH on TUESDAY NIGHT, the 25th MARCH. Advertisements intended for insertion in this issue should therefore reach us by 4 p.m. ON TUESDAY, the 25th MARCH.**

### NOTICE TO CORRESPONDENTS.

All MSS., Letters, Books for Review, and other matters intended for the Editor should be addressed The Editor "The Hospital," The Hospital Building, 28 & 29 Southampton Street, Strand, W.C.

The Editor cannot undertake to return rejected MS., even when accompanied by stamped directed envelope.

### Notice to Advertisers and Subscribers.

All Advertisements, Orders for Copies of The Hospital, and Business Communications should be addressed to **THE MANAGER, (Not to the Editor)**, The Hospital, 28 & 29 Southampton Street, Strand, London, W.C.

The Cost of Subscription, post free, is as follows.—Per week, 2½d.; per quarter, 2s. 8d; per half-year, 5s. 3d.; per annum, 10s. 6d. Foreign:—Per annum, 15s 2d. payable, in all cases, in advance.

**NOTICE.—Vol. XXX. of "The Hospital" (April 1901, to September 1901), in handsome cloth binding can now be obtained at the office of this Journal, price, 7s. 6d. Cases for binding the Numbers contained in this Volume 1s. 6d. Index to Vol. XXX., 2½d., post free.**

**The Monthly Part for March is now ready. Price 6d., by post 8d.**

### SCRAPS AND GLEANINGS.

A concert (under royal patronage) is to be given on June 2nd in aid of the Ladies' Association of the Metropolitan Hospital, Kingsland Road.

During the past year the staff of the Maternity Charity and District Nurses' Home, Plaistow, East Ham, and the Docks, have nursed 5,949 patients in their own homes, which necessitated 139,149 visits. This is the largest number of patients ever treated in one year being 800 in excess of the previous record.

### New Works for Nurses.

### LESSONS ON MASSAGE.

By MRS. MARGARET D. PALMER, Manager of the Massage Department and Instructor of Massage to the Nursing Staff of the London Hospital. 258 pages, with 106 Illustrations, mostly original. Price 7/6 net.

### A MANUAL FOR STUDENTS OF MASSAGE.

By MISS M. A. ELLISON, L.O.S. Price 3/6 net. With 50 original Illustrations.

### COMMONWEALTH of CELLS.

**Being Essays on Elementary Physiology.**

By H. G. F. SPURRELL, M.A. Oxon. With 67 original Illustrations. 2/6 net.

" Nothing has been written so likely to popularise the fascinating subject of physiology as these essays by Mr. Spurrell."—*Lancet.*

### MENSTRUATION AND ITS DISORDERS.

By ARTHUR E. GILES, M.D., B.Sc., F.R.C.S., Surgeon Chelsea Hospital for Women. Just out. Price 2/6 net.

### MOVABLE ATLAS OF THE ANATOMY AND PHYSIOLOGY OF THE FEMALE GENERATIVE ORGANS AND OF PREGNANCY.

With Explanatory Text. Price 3/- net. By ARTHUR E. GILES, M.D., F.R.C.S.

### MOVABLE ATLAS OF THE ANATOMY AND PHYSIOLOGY OF THE CHILD.

With Explanatory Text. By D'ARCY POWER, M.A.Oxon, M.B., F.R.C.S., Surgeon Victoria Hospital for Children; Assistant Surgeon St. Bartholomew's Hospital. Price 3/- net.

London: BAILLIÈRE, TINDALL & COX, 8 Henrietta Street, Covent Garden, W.C.

Post 8vo. pp. xv.-245, and 63 illustrations in the text. Cash price, 4s. 6d.; postage, 4d.

### PRACTICAL TEXT-BOOK OF MIDWIFERY FOR NURSES AND STUDENTS,

By ROBERT JARDINE, M.D., Edin., M.R.C.S., Eng., F.F.P. and S., Glas.,

*Physician to the Glasgow Maternity Hospital.*

" As a work for nurses only we shall have no hesitation in saying that [illegible] is the best suited for its purpose of any book of the kind which we ha[illegible] come across."—*The Dublin Journal of Medical Science.*

W. F. CLAY, Publisher, Edinburgh.

